# Supplementary material for: A health terminological system for inherited retinal diseases: Content coverage evaluation and a proposed classification
Source: PLoS One. 2023 Aug 4;18(8):e0281858. doi: 10.1371/journal.pone.0281858 (PMC10403057; doi:10.1371/journal.pone.0281858)
Supplement: S1 Table — (DOCX) [file pone.0281858.s002.docx]

| **Synonym/ Alternative Concept** | **IRD Concepts** | **Internal Code**  Table 1. A preliminary classification of inherited retinal dystrophy (IRD) concepts. |
| --- | --- | --- |
| **Diffuse Photoreceptor Dystrophy** | | 1. |
| RP | Retinitis pigmentosa | 1.1. |
| - | Typical retinitis pigmentosa | 1.1.1. |
| Pericentral retinitis pigmentosa | Central retinitis pigmentosa | 1.1.2. |
| - | Retinitis punctata albescens | 1.1.3. |
| Regional retinitis pigmentosa | Sector retinitis pigmentosa | 1.1.4. |
| Infantile to early childhood retinitis pigmentosa | Leber congenital amaurosis | 1.2. |
| Cone-rod dystrophy | | 1.3. |
| **Macular Dystrophies** | | 2. |
| Juvenile macular dystrophy | Stargardt disease and fundus flavimaculatus | 2.1. |
| - | Vitelliform degenerations | 2.2. |
| Best vitelliform dystrophy | Best disease | 2.2.1. |
| - | Adult-onset vitelliform lesions | 2.2.2. |
| - | Familial (dominant) drusen | 2.3. |
| - | Pattern dystrophies | 2.4. |
| - | Sorsby macular dystrophy | 2.5. |
| **Chorioretinal Dystrophies** | | 3. |
| - | Diffuse degeneration of choroid | 3.1. |
| - | [Choroideremia (degeneration of retinal pigmented epithelium + choriocapillaris)](https://www.google.com/search?biw=1366&bih=662&q=Choroideremia+(degeneration+of+RPE+%2B+choriocapillaris)&spell=1&sa=X&ved=0ahUKEwj7ks3umKjSAhUF7xQKHZ9lBrgQvwUIFigA) | 3.1.1. |
| - | Gyrate atrophy | 3.1.2. |
| - | Regional choroidal dystrophies | 3.2. |
| - | Benign concentric annular macular dystrophy | 3.2.1. |
| - | North carolina macular dystrophy | 3.2.2. |
| CACD | Central areolar choroidal dystrophy | 3.2.3. |
| **Inner Retinal and Vitreoretinal Dystrophies** | | 4. |
| - | X-linked retinoschisis | 4.1. |
| - | Goldmann-Favre syndrome | 4.2. |
| **Systemic Diseases Associated with Photoreceptor Dystrophies** | | 5. |
| [Abetalipoproteinemia](https://www.google.com/search?biw=1366&bih=662&q=1.%09Bassen-Kornzweig+Syndrome+(abetalipoproteinemia)&spell=1&sa=X&ved=0ahUKEwiIx930jKjSAhWLOBQKHUfCALgQvwUIFigA) | Bassen-Kornzweig syndrome | 5.1. |
| - | Refsum disease | 5.2. |
| - | [Kearns-Sayre syndrome](https://www.google.com/search?biw=1366&bih=662&q=3.%09Kearns-Sayre+Syndrome&spell=1&sa=X&ved=0ahUKEwik4LSKjajSAhVF7RQKHdTTDbcQvwUIFigA) | 5.3. |
| - | [Bardet-Biedl syndrome](https://www.google.com/search?biw=1366&bih=662&q=4.%09Bardet-Biedl+Syndrome&spell=1&sa=X&ved=0ahUKEwjNuKiWjajSAhXG7BQKHbXmDLsQvwUIFigA) | 5.4. |
| - | Usher syndrome | 5.5. |
| [**Congenital & Stationary Retinal Disease**](http://scholar.google.com/scholar?q=Congenital+%26+Stationary+Retinal+Disease&hl=en&as_sdt=0&as_vis=1&oi=scholart&sa=X&ved=0ahUKEwjh9dL0lKjSAhUFuRoKHZC9CqsQgQMIFzAA)**s** | | 6. |
| - | With normal fundus + no retinal dystrophy | 6.1. |
| **-** | Color vision abnormalities (cone system) | 6.1.1. |
| CSNB | Congenital stationary night-blinding disease with normal fundi | 6.1.2. |
| **-** | Schubert- Bornschein form of CSNB | 6.1.2.1 |
| - | Complete type | 6.1.2.1.1. |
| - | Incomplete type | 6.1.2.1.2. |
| - | Reduction of both scotopic a- and scotopic b- waves | 6.1.2.2. |
| - | Abnormal fundus + no retinal dystrophy (disorders in metabolism of photoreceptors) | 6.2. |
| - | Congenital night- blinding disorders with prominent fundus abnormality | 6.2.1. |
| - | Fundus albipunctatus | 6.2.1.1. |
| - | Oguchi disease | 6.2.1.2. |
| - | Abnormal fundus + retinal dystrophy | 6.3. |
| Atypical Achromatopsia | Blue cone monochromatism | 6.3.1. |
| - | Congenital color deficiency | 6.4. |
| **-** | Hereditary | 6.4.1. |
| **-** | Trichromatism | 6.4.1.1. |
| **-** | Deutranomalous | 6.4.1.1.1. |
| **-** | Protanomalous | 6.4.1.1.2. |
| **-** | Tritanomalous | 6.4.1.1.3. |
| **-** | Dichromatism | 6.4.1.2. |
| **-** | Deutranopia | 6.4.1.2.1. |
| **-** | Protanopia | 6.4.1.2.2. |
| **-** | Tritanopia | 6.4.1.2.3. |
| Monochromatism | Achromatopsia | 6.4.1.3. |
| Rod monochromatism | Typical | 6.4.1.3.1. |
| **-** | Acquired | 6.4.1.4. |
| **-** | Tritan (blue-yellow) | 6.4.1.4.1. |
| **-** | Protan- deutran (red- green) | 6.4.1.4.2. |
