# Supplementary material for: A health terminological system for inherited retinal diseases: Content coverage evaluation and a proposed classification
Source: PLoS One. 2023 Aug 4;18(8):e0281858. doi: 10.1371/journal.pone.0281858 (PMC10403057; doi:10.1371/journal.pone.0281858)
Supplement: S2 Table — (DOCX) [file pone.0281858.s003.docx]

Table 2. Not validated concepts as inherited retinal dystrophy (IRD).

| **No** | **Concepts** |
| --- | --- |
| 1 | Albers-Schönberg osteopetrosis |
| 2 | Foveal hypoplasia- presenile cataract syndrome |
| 3 | Hermansky- Pudlak syndrome with neutropenia |
| 4 | Leigh syndrome |
| 5 | Metachromatic leukodystrophy |
| 6 | Rubinstein- Taybi syndrome |
| 7 | Retinal vasculopathy with cerebral leukonecephalopathy and systemic manifestation |
| 8 | Revesz syndrome |
| 9 | Aceruloplasminemia |
| 10 | Cockayne syndrome |
| 11 | Xeroderma pigmentosum- cockayne syndrome complex |
