# Supplementary material for: A health terminological system for inherited retinal diseases: Content coverage evaluation and a proposed classification
Source: PLoS One. 2023 Aug 4;18(8):e0281858. doi: 10.1371/journal.pone.0281858 (PMC10403057; doi:10.1371/journal.pone.0281858)
Supplement: S3 Table — (DOCX) [file pone.0281858.s004.docx]

| **Our Proposed Ontology** | **Applied Terminological System** | **IRD Concept** | **No**  Table 3. Classification of IRD concepts in our proposed ontology and applied standard terminological system. |
| --- | --- | --- | --- |
| Systemic/ Syndromic Diseases Associated with Retinal Dystrophies | Genetic Macular Dystrophy | AICA- ribosiduria | 1 |
| Chorioretinal Dystrophies | Genetic Macular Dystrophy | Benign concentric annular macular dystrophy | 2 |
| Systemic/ Syndromic Diseases Associated with Retinal Dystrophies | Genetic Macular Dystrophy | EEM syndrome | 3 |
| Dystrophies Primarily Involving the Retinal Pigment Epithelium | Genetic Macular Dystrophy | Patterned dystrophy of retinal pigmented epithelium * | 4 |
| Diffuse Photoreceptor Dystrophies | Genetic Macular Dystrophy | Progressive cone dystrophy | 5 |
| Chorioretinal Dystrophies | Genetic Macular Dystrophy | Bietti crystalline dystrophy ** | 6 |
| Congenital & Stationary Retinal Diseases | Genetic Macular Dystrophy | Fundus albipunctatus | 7 |
| Diffuse Photoreceptor Dystrophies | Genetic Macular Dystrophy | Retinitis punctate albescens | 8 |
| Dystrophies Primarily Involving the Retinal Pigment Epithelium | Genetic Macular Dystrophy | Butterfly- shaped pigment dystrophy | 9 |
| Dystrophies Primarily Involving the Retinal Pigment Epithelium | Genetic Macular Dystrophy | Fundus pulverulentus | 10 |
| Dystrophies Primarily Involving the Retinal Pigment Epithelium | Genetic Macular Dystrophy | Multifocal pattern dystrophy simulating fundus flavimaculatus | 11 |
| Dystrophies Primarily Involving the Retinal Pigment Epithelium | Genetic Macular Dystrophy | Reticular dystrophy of the retinal pigmented epithelium | 12 |
| Systemic/ Syndromic Diseases Associated with Retinal Dystrophies | Genetic Macular Dystrophy | Bardet- Biedl syndrome | 13 |
| Diffuse Photoreceptor Dystrophies | Genetic Macular Dystrophy | Cone- rod dystrophy | 14 |
| Inner Retinal and/or Vitreoretinal Dystrophies | Genetic Macular Dystrophy | Goldmann- Farve syndrome | 15 |
| Systemic/ Syndromic Diseases Associated with Retinal Dystrophies | Genetic Macular Dystrophy | Hypotrichosis with juvenile macular degeneration | 16 |
| Systemic/ Syndromic Diseases Associated with Retinal Dystrophies | Genetic Macular Dystrophy | Infantile Refsum disease | 17 |
| Systemic/ Syndromic Diseases Associated with Retinal Dystrophies | Genetic Macular Dystrophy | Laurence- Moon syndrome | 18 |
| Systemic/ Syndromic Diseases Associated with Retinal Dystrophies | Genetic Macular Dystrophy | Sjögren- Larsson syndrome | 19 |
| Inner Retinal and/or Vitreoretinal Dystrophies | Genetic Macular Dystrophy | X- linked retinoschisis | 20 |
| Systemic/ Syndromic Diseases Associated with Retinal Dystrophies | Genetic Macular Dystrophy | Zellweger syndrome | 21  IRD, Inherited retinal dystrophy; AICA, 5-amino-4-imidazole carboxamide; EEM, Ectodermal dysplasia, ectrodactyly and macular dystrophy  * Labelled as "patterned dystrophy of macula" in our proposed ontology.  **Labelled as "Bietti crystalline retinopathy" in our proposed ontology. |
