# Supplementary material for: A health terminological system for inherited retinal diseases: Content coverage evaluation and a proposed classification
Source: PLoS One. 2023 Aug 4;18(8):e0281858. doi: 10.1371/journal.pone.0281858 (PMC10403057; doi:10.1371/journal.pone.0281858)
Supplement: S4 Table — (DOCX) [file pone.0281858.s005.docx]

| **No**  Table 4. A list of IRD concepts coded by all systems and those coded by none of systems. | **Internal Code** | **IRD Concepts Coded by All Systems** |
| --- | --- | --- |
| 1 | IRD_1_1 | Retinitis pigmentosa |
| 2 | IRD_1_1_1 | Retinitis punctata albescens |
| 3 | IRD_1_1_4 | Late- onset retinal degeneration; LORD |
| 4 | IRD_1_5_2 | Cone dystrophy with supernormal rod response |
| 5 | IRD_1_6 | Bothnia retinal dystrophy |
| 6 | IRD_2_1_2 | Fundus flavimaculatus |
| 7 | IRD_2_2_6 | Kandori fleck retina |
| 8 | IRD_2_3_2 | Reticular dystrophy of the retinal pigment epithelium |
| 9 | IRD_2_6 | Occult macular dystrophy; OCMD |
| 10 | IRD_3_1_1 | Helicoid peripapillary chorioretinal degeneration; HPCD |
| 11 | IRD_3_1_2 | Choroideremia; CHM |
| 12 | IRD_3_1_4 | Gyrate atrophy; GACR |
| 13 | IRD_3_1_6 | Bietti crystalline retinopathy; BCD |
| 14 | IRD_3_2_1 | Benign concentric annular macular dystrophy |
| 15 | IRD_3_2_2 | North Carolina macular dystrophy |
| 16 | IRD_3_2_4 | Progressive bifocal chorioretinal atrophy |
| 17 | IRD_4_2_2 | Pigmented paravenous retinochoroidal atrophy; PPCRA |
| 18 | IRD_4_1 | X-linked retinoschisis |
| 19 | IRD_4_2 | Enhanced S cone syndrome (Goldmann-Favre syndrome) |
| 20 | IRD_5_2 | Alstrom syndrome |
| 21 | IRD_5_7 | Bassen-Kornzweig syndrome |
| 22 | IRD_5_12 | Cohen syndrome; COH1 |
| 23 | IRD_5_13 | Coloboma of macula with type B brachydactyly (Sorsby syndrome) |
| 24 | IRD_5_16 | EEM syndrome (Ectodermal dysplasia, ectrodactyly, and macular dystrophy) |
| 25 | IRD_5_19 | Farber disease |
| 26 | IRD_5_20 | Galactosialidosis; GSL |
| 27 | IRD_5_21_2 | GM1 gangliosidosis, type II |
| 28 | IRD_5_21_3 | GM1 gangliosidosis, type III |
| 29 | IRD_5_23 | Hurler syndrome |
| 30 | IRD_5_24 | Hurler-Scheie syndrome |
| 31 | IRD_5_27 | Hypotrichosis with juvenile macular degeneration; HJMD |
| 32 | IRD_5_29_1 | ARIMA syndrome |
| 33 | IRD_5_30 | Kearns-Sayre syndrome |
| 34 | IRD_5_31 | Krabbe disease |
| 35 | IRD_5_34 | McKusick Kaufman syndrome; MKKS |
| 36 | IRD_5_36 | Microcephaly-lymphedema-chorioretinopathy syndrome |
| 37 | IRD_5_40 | MORM syndrome (mental retardation, truncal obesity, retinal dystrophy and micropenis) |
| 38 | IRD_5_42 | Mucolipidosis type IV; ML4 |
| 39 | IRD_5_44 | Neuropathy, ataxia, and retinitis pigmentosa (NARP syndrome) |
| 40 | IRD_5_46 | Oculotrichodysplasia |
| 41 | IRD_5_50_2 | Neonatal adrenoleukodystrophy |
| 42 | IRD_5_55_2 | Infantile Refsum disease |
| 43 | IRD_5_56 | Retinal degeneration- nanophthalmos- glaucoma syndrome |
| 44 | IRD_5_66 | RHYNS syndrome |
| 45 | IRD_5_67 | Scheie syndrome |
| 46 | IRD_5_70 | Sjögren-Larsson syndrome; SLS |
| 47 | IRD_6_1_2_1 | Aland Island eye disease; AIED |
| 48 | IRD_6_2_1 | Fundus albipunctatus |
| 49 | IRD_6_2_3 | X-linked cone dysfunction syndrome with myopia (Bornholm Eye Disease) |
| 50 | IRD_6_3_2 | Blue cone monochromatism; BCM |
| 51 | IRD_6_3_3 | Bornholm Eye Disease (X-Linked Cone Dysfunction Syndrome With Dichromacy) |
| **IRD Concepts Coded by None of Systems** | | |
| 1 | IRD_5_69 | Short- rib thoracic dysplasia with or without polydactyly; SRTD |
| 2 | IRD_6_1_2 | Congenital stationary night blindness with normal fundus, known incomplete type |
| 3 | IRD_6_3 | Disorders of the cone system |
