# Supplementary material for: A health terminological system for inherited retinal diseases: Content coverage evaluation and a proposed classification
Source: PLoS One. 2023 Aug 4;18(8):e0281858. doi: 10.1371/journal.pone.0281858 (PMC10403057; doi:10.1371/journal.pone.0281858)
Supplement: Attachment — Submitted filename: Response to Reviewers.docx [file pone.0281858.s006.docx]

PONE-D-22-12220

A Health Terminological System for Inherited Retinal Diseases: Content Coverage Evaluation and a Proposed Novel Classification

PLOS ONE

Dear Dr. Sheikhtaheri,

Thank you for submitting your manuscript to PLOS ONE. After careful consideration, we feel that it has merit but does not fully meet PLOS ONE’s publication criteria as it currently stands. Therefore, we invite you to submit a revised version of the manuscript that addresses the points raised during the review process. I am sorry that it has taken a long time to present you with a review, but we were trying to find a second expert to review your submission.  In the interests of time, I decided to ask for revision based on the comments of one expert whose comments are valuable.

Please submit your revised manuscript by Sep 01 2022 11:59PM. If you will need more time than this to complete your revisions, please reply to this message or contact the journal office at [plosone@plos.org](mailto:plosone@plos.org). When you're ready to submit your revision, log on to <https://www.editorialmanager.com/pone/> and select the 'Submissions Needing Revision' folder to locate your manuscript file.

Please include the following items when submitting your revised manuscript:

- A rebuttal letter that responds to each point raised by the academic editor and reviewer(s). You should upload this letter as a separate file labeled 'Response to Reviewers'.
- A marked-up copy of your manuscript that highlights changes made to the original version. You should upload this as a separate file labeled 'Revised Manuscript with Track Changes'.
- An unmarked version of your revised paper without tracked changes. You should upload this as a separate file labeled 'Manuscript'.

If you would like to make changes to your financial disclosure, please include your updated statement in your cover letter. Guidelines for resubmitting your figure files are available below the reviewer comments at the end of this letter.

If applicable, we recommend that you deposit your laboratory protocols in protocols.io to enhance the reproducibility of your results. Protocols.io assigns your protocol its own identifier (DOI) so that it can be cited independently in the future. For instructions see: <https://journals.plos.org/plosone/s/submission-guidelines#loc-laboratory-protocols>. Additionally, PLOS ONE offers an option for publishing peer-reviewed Lab Protocol articles, which describe protocols hosted on protocols.io. Read more information on sharing protocols at <https://plos.org/protocols?utm_medium=editorial-email&utm_source=authorletters&utm_campaign=protocols>.

We look forward to receiving your revised manuscript.

Kind regards,

Alfred S Lewin, Ph.D.
Section Editor
PLOS ONE

Journal Requirements:

When submitting your revision, we need you to address these additional requirements.

1. Please ensure that your manuscript meets PLOS ONE's style requirements, including those for file naming. The PLOS ONE style templates can be found at

<https://journals.plos.org/plosone/s/file?id=wjVg/PLOSOne_formatting_sample_main_body.pdf>  and

<https://journals.plos.org/plosone/s/file?id=ba62/PLOSOne_formatting_sample_title_authors_affiliations.pdf>

2. Thank you for stating the following financial disclosure:

“The funders had no role in study design, data collection and analysis, decision to publish, or preparation of the manuscript.”

At this time, please address the following queries:

a)        Please clarify the sources of funding (financial or material support) for your study. List the grants or organizations that supported your study, including funding received from your institution.

b)        State what role the funders took in the study. If the funders had no role in your study, please state: “The funders had no role in study design, data collection and analysis, decision to publish, or preparation of the manuscript.”

c)        If any authors received a salary from any of your funders, please state which authors and which funders.

d)        If you did not receive any funding for this study, please state: “The authors received no specific funding for this work.”

Please include your amended statements within your cover letter; we will change the online submission form on your behalf.

3. Please include captions for your Supporting Information files at the end of your manuscript, and update any in-text citations to match accordingly. Please see our Supporting Information guidelines for more information: <http://journals.plos.org/plosone/s/supporting-information>.

[Note: HTML markup is below. Please do not edit.]

Editor comment

Please modify your comments about the novelty of your work, because there are already several classification systems for IRDs, though none is widely accepted. Please explain how genetic data informed your classification scheme.  The reviewer indicates that including genotypic information is important.

**Answer:** The IRD hierarchy has been revised based on both genetic and anatomical entities. Also, all required modifications have been performed throughout the text. Although there are several classification systems for rare eye diseases, no certain hierarchy has been developed specifically for IRD concepts. The current IRD classification has been developed based on the textbooks review, and the related standard terminologies such as Unified Medical Language System (UMLS), Online Mendelian Inheritance in Man (OMIM), International Classification of Diseases (ICD-11), Systematized Nomenclature of Medicine (SNOMED-CT) and Orphanet Rare Disease Ontology (ORDO) which is more comprehensive compared with other classifications.

Reviewers' comments:

Reviewer's Responses to Questions

**Comments to the Author**

1. Is the manuscript technically sound, and do the data support the conclusions?

The manuscript must describe a technically sound piece of scientific research with data that supports the conclusions. Experiments must have been conducted rigorously, with appropriate controls, replication, and sample sizes. The conclusions must be drawn appropriately based on the data presented.

Reviewer #1: Partly

**Answer:** The conclusion part has been revised to be completely in line with the study findings.

2. Has the statistical analysis been performed appropriately and rigorously?

Reviewer #1: N/A

3. Have the authors made all data underlying the findings in their manuscript fully available?

The [PLOS Data policy](http://www.plosone.org/static/policies.action#sharing) requires authors to make all data underlying the findings described in their manuscript fully available without restriction, with rare exception (please refer to the Data Availability Statement in the manuscript PDF file). The data should be provided as part of the manuscript or its supporting information, or deposited to a public repository. For example, in addition to summary statistics, the data points behind means, medians and variance measures should be available. If there are restrictions on publicly sharing data—e.g. participant privacy or use of data from a third party—those must be specified.

Reviewer #1: Yes

4. Is the manuscript presented in an intelligible fashion and written in standard English?

PLOS ONE does not copyedit accepted manuscripts, so the language in submitted articles must be clear, correct, and unambiguous. Any typographical or grammatical errors should be corrected at revision, so please note any specific errors here.

Reviewer #1: Yes

5. Review Comments to the Author

Please use the space provided to explain your answers to the questions above. You may also include additional comments for the author, including concerns about dual publication, research ethics, or publication ethics. (Please upload your review as an attachment if it exceeds 20,000 characters)

Reviewer #1: The authors present a novel IRD classification. The methodology used to achieve this new classification is well defined and all data was made available. Please find my queries below:

Abstract

– Purpose: Please use “To present a novel…” instead of “To represent a novel…”

**Answer:** It has been done.

Introduction

– The authors write: “to the best of our knowledge there is no dedicated ontology or classification for IRD concepts”. This is not true. There are several proposed classifications. However, I would say there is not one widely accepted classification. Also, please check PMID: 30626441 (ref 8 in your references list). The authors present a summary of the modifications made to HPO and ORDO as part of the study (Table S1), and list the disease groups and specific disorders introduced to ORDO as a result of the ERN-EYE Ontology meeting (Mont Sainte-Odile, France, 10/2017). The ERN-EYE is an European Reference Network covering all rare eye disorders and not only IRDs. The group has worked hand in hand with ORPHANET to improve IRD and other rare eye disease nomenclature in ORDO.

**Answer:** Although there are several classification systems for rare eye diseases, no certain hierarchy has been developed specifically for IRD concepts. The current IRD classification has been developed based on the text books review, and the related standard terminologies such as Unified Medical Language System (UMLS), Online Mendelian Inheritance in Man (OMIM), International Classification of Diseases (ICD-11), Systematized Nomenclature of Medicine (SNOMED-CT) and Orphanet Rare Disease Ontology (ORDO) which is more comprehensive compared with other classifications.

– Please replace “…to validate the value of this novel…” for “…to validate this novel…”

**Answer:** It has been done.

Methods

– Step I: the authors write “This proposed classification was structured based on the phenotype characteristics and the genetic concepts were included to the related IRD diagnosis.” Please elaborate on the genetic concepts. I cannot find in the text, tables, figures or supplemental material any reference to the genetic cause of the proposed diagnoses. In fact, I would say the major issue with the presented classification is that it lacks genetic information. Please elaborate on this as it is a fundamental part of any new IRD classification. Several genes are able to cause different phenotypes and gene-based classifications are getting more popular. Developing a strong phenotypical classification is important but it must incorporate genetic data, ie, genes associated with each phenotype and must allow seeing things the other way around, ie which phenotypes are associated with each gene.

**Answer:** The IRD hierarchy has been revised based on both genetic and anatomical entities. Also, all required modifications have been performed throughout the text.

– Another issue is that some diagnoses fit more than one category. An example would be central areolar choroidal dystrophy (CACD) which fits the macular dystrophies group and choroidal dystrophies group. The same is true for syndromic forms of retinitis pigmentosa (eg Usher, BBS) that fit the Retinal dystrophies associated with systemic disease (syndromic diseases) group and the Diffuse Photoreceptor Dystrophies group; or syndromic forms of cone-rod dystrophy (eg. Cone-rod dystrophy and hearing loss, caused by CEP250 mutations) that fit the Retinal dystrophies associated with systemic disease (syndromic diseases) group Diffuse Photoreceptor Dystrophies group. Please comment.

**Answer:** Thank you for raising this concern, which is a potential issue in any classification system. In our proposed classification system, each IRD disease is mainly grouped based on the main origin of its pathological problem considering both anatomical and functional concepts. Although, many IRDs gradually progress and after a while will involve other adjacent structures. For instance, as you indicated CACD is mostly a choroidal disease that gradually extends to involve the macular region or farther parts of the retina. Therefore, in our classification system, we grouped this diagnosis under choroidal dystrophies. In the same way, we classified the “diffuse photoreceptor dystrophies” as a group containing the IRDs that exclusively involve the retina with no other organ involvement, besides there is another group of “systemic diseases associated with photoreceptor dystrophies” to embrace retinal dystrophies that have an associated systemic or organ condition such as BBS and Usher syndrome or cone-rod dystrophy and hearing loss. Please refer to supplementary table 1.

– What about cases where the phenotype changes or does not fit any of the proposed groups? Some PRPH2-associated macular dystrophies for instance. Shouldn’t there be a group for cases that cannot be classified anywhere else?

**Answer:** You have pointed out a great example. We reported a complex family affected with different clinical features of inherited retinal dystrophy, including fundus flavimaculatus as an early clinical feature progressing to extensive chorioretinal atrophy involving the macula and mid-periphery of the fundus in one parent and central areolar chorioretinal dystrophy (CACD) as the most probable clinical diagnosis in another parent. Macular pattern dystrophy for one of their daughters and a Leber congenital amaurosis (LCA) like phenotype for the daughter with an early onset retinal dystrophy (EORD) phenotype. (Daftarian N, et al. Ophthalmic Genetics, 40:5, 436-442). Therefore, in our proposed classification, the disease categorization was organized considering both anatomical and functional IRD entities in seven groups, instead of being exclusively based on the genetic, anatomic or functional diagnosis. Supplementary table 1

– Is the term “regional retinitis pigmentosa” a synonym of “sector retinitis pigmentosa”? If yes, then the term sector retinitis pigmentosa should be used instead, as this is the most common term.

**Answer:** Thank you for alluding to another precise point. We replaced it with the more familiar term “sector retinitis pigmentosa” and then will use the regional retinitis pigmentosa as the alternative one.

References

– Duplicate references! Ref 8 and ref 21 are the same.

**Answer:** Many thanks, the list of references has been revised.

6. PLOS authors have the option to publish the peer review history of their article. If published, this will include your full peer review and any attached files.
If you choose “no”, your identity will remain anonymous but your review may still be made public.


**Do you want your identity to be public for this peer review?** For information about this choice, including consent withdrawal, please see our [Privacy Policy](https://www.plos.org/privacy-policy).

Reviewer #1: No
