# Supplementary material for: A health terminological system for inherited retinal diseases: Content coverage evaluation and a proposed classification
Source: PLoS One. 2023 Aug 4;18(8):e0281858. doi: 10.1371/journal.pone.0281858 (PMC10403057; doi:10.1371/journal.pone.0281858)
Supplement: Attachment — Submitted filename: Response to Reviewers.docx [file pone.0281858.s007.docx]

PONE-D-22-12220R1

A Health Terminological System for Inherited Retinal Diseases: Content Coverage Evaluation and a Proposed Classification

PLOS ONE


Editor comment

Please provide a table in which the genes associated with each proposed diagnosis are shown. Your final classification system should contain both anatomical and genetic classifications.

Thank you for the comment. We added genes information.

Reviewers' comments:

Reviewer's Responses to Questions

**Comments to the Author**

1. If the authors have adequately addressed your comments raised in a previous round of review and you feel that this manuscript is now acceptable for publication, you may indicate that here to bypass the “Comments to the Author” section, enter your conflict of interest statement in the “Confidential to Editor” section, and submit your "Accept" recommendation.

Reviewer #1: (No Response)

2. Is the manuscript technically sound, and do the data support the conclusions?

The manuscript must describe a technically sound piece of scientific research with data that supports the conclusions. Experiments must have been conducted rigorously, with appropriate controls, replication, and sample sizes. The conclusions must be drawn appropriately based on the data presented.

Reviewer #1: Partly

**Answer:** The conclusion part has been revised to be more corresponding with our findings.

3. Has the statistical analysis been performed appropriately and rigorously?

Reviewer #1: N/A

4. Have the authors made all data underlying the findings in their manuscript fully available?

The [PLOS Data policy](http://www.plosone.org/static/policies.action#sharing) requires authors to make all data underlying the findings described in their manuscript fully available without restriction, with rare exception (please refer to the Data Availability Statement in the manuscript PDF file). The data should be provided as part of the manuscript or its supporting information, or deposited to a public repository. For example, in addition to summary statistics, the data points behind means, medians and variance measures should be available. If there are restrictions on publicly sharing data—e.g. participant privacy or use of data from a third party—those must be specified.

Reviewer #1: Yes

5. Is the manuscript presented in an intelligible fashion and written in standard English?

PLOS ONE does not copyedit accepted manuscripts, so the language in submitted articles must be clear, correct, and unambiguous. Any typographical or grammatical errors should be corrected at revision, so please note any specific errors here.

Reviewer #1: Yes

6. Review Comments to the Author

Please use the space provided to explain your answers to the questions above. You may also include additional comments for the author, including concerns about dual publication, research ethics, or publication ethics. (Please upload your review as an attachment if it exceeds 20,000 characters)

Reviewer #1: Thank you for your revision. You have addressed most of my concerns. However, one major issue remains:

I still cannot find any table or supplemental table where the genes associated with each proposed diagnosis are shown. The link you provide with your final classification (<https://bioportal.bioontology.org/ontologies/IRD1)> separates the genetic classification (genetic_variantion) from the anatomical classification (Inherited_retinal_Dystrophy). It is my understanding that a novel classification should merge both.
Let’s see a practical example: retinitis pigmentosa associated with biallelic EYS variants:

According to your proposed classification system, this would be:
IRD_1_1 but there are several causative genes so we should be able to select the causative gene (IRD_1_1#21 according to your genetic variation list) from the first classification.

Please provide a merged classification.

**Answer:** The merged IRDs hierarchy combining both anatomical and genetic codes providing based on five standard coding systems has been submitted to the website as well as the BioPortal website.


Minor issues

- Why do you separate AR and AD pericentral pigmentary retinopathy? I understand the same phenotype may be caused by AR or AD genes but if you are separating it here according to the inheritance pattern, then you should be doing the same for Retinitis Pigmentosa (AR, AD, XL), Cone-rod dystrophy (AR, AD, XL), etc.

**Answer:** The title of each IRD entity either includes the inheritance pattern or not has been selected based on the standard terminologies without any updates and no suggested terms have been inserted into the [heicharcy](https://www.google.com/search?rlz=1C1GCEA_enIR822IR822&sxsrf=AJOqlzWZzz2gX5uR1zDx2rZCrAeqnOtHug:1673767345377&q=heicharcy&spell=1&sa=X&ved=2ahUKEwiz_bqghcn8AhXxhf0HHbNuAt0QkeECKAB6BAgIEAE).

- Wouldn’t it be easier to join Choroidal dystrophies (IRD 3) with Chorioretinal dystrophies (IRD 7)?

**Answer:** These two categories have been merged into the one group labeled as "Choroidal Dystrophy".

- Regarding the "Supporting Information – Final Classification and Coverage Version 12.18.2021", the authors did not change the term “regional” to “sector” retinitis pigmentosa.

**Answer:** It has been changed as you recommended.

7. PLOS authors have the option to publish the peer review history of their article ([what does this mean?](https://journals.plos.org/plosone/s/editorial-and-peer-review-process#loc-peer-review-history)). If published, this will include your full peer review and any attached files.


If you choose “no”, your identity will remain anonymous but your review may still be made public.


**Do you want your identity to be public for this peer review?** For information about this choice, including consent withdrawal, please see our [Privacy Policy](https://www.plos.org/privacy-policy).

Reviewer #1: No
